# Shared genetic features inference among hypoxia-ischemia diseases in the presence of heterogenous omics data based on a novel risk assessment method

**DOI:** 10.3389/fgene.2025.1587854

**Published:** 2025-04-28

**Authors:** Yifan Zhang, Jianfeng Liu, Zhuoma Basang, Qianxun Yang, Hongce Chen, Shuo Chen, Shaogang Li, Changgui Lei, Mingyan Fang, Huanhuan Liu, Xin Jin, Yingying Wang

**Affiliations:** ^1^ BGI Research, Chongqing, China; ^2^ BGI Research, Shenzhen, China; ^3^ College of Life Sciences, University of Chinese Academy of Sciences, Beijing, China; ^4^ Shenzhen Key Laboratory of Transomics Biotechnologies, BGI Research, Shenzhen, China; ^5^ Department of Neurology, The Eighth Affiliated Hospital, Sun Yat-Sen University, Shenzhen, China; ^6^ High Altitude Health Science Research Center, Tibet University, Lhasa, Tibet, China; ^7^ College of Life Sciences and Oceanography, Shenzhen University, Shenzhen, Guangdong, China; ^8^ School of Life Sciences, Lanzhou University, Lanzhou, Gansu, China; ^9^ College of Plant Protection, Hunan Agricultural University, Changsha, Hunan, China; ^10^ School of Biology and Biological Engineering, South China University of Technology, Guangzhou, China; ^11^ BGI Research, Wuhan, China; ^12^ State Key Laboratory of Genome and Multi-omics Technologies, BGI Research, Shenzhen, China

**Keywords:** risk assessment, hypoxia-ischemia, shared features, disease profile, omics

## Abstract

The hypoxia-ischemia (H-I) diseases share some common mechanisms which may help to delay the diseases’ processing. However, the shared features are still unclear due to the lack of large scale high-quality multi - omics data that specifically target the same disease, population, and tissues/cells. In this study, we developed a novel risk assessment method to analyze four H–I diseases including eclampsia/preeclampsia (PE), pulmonary arterial hypertension (PAH), high-altitude polycythemia (HAPC), and ischemic stroke (IS). A combined new evaluation score was designed to integrate evaluation information from genomics, transcriptomics, proteomics, and metabolomics in previous researches. Genes were then divided into different groups according to their risk assessment score. The most significant group (direct biomarkers) contained genes with direct evidence of association to H-I disease: *PIEZO2* and *HPGD* (shared), *TSIX* and *SAA1* (PAH - specific), *GSTM1*, *DNTT*, and *IGKC* (HAPC - specific), *LEP*, *SERPINA3*, and *ARHGEF4* (PE - specific), *CD3D*, *ITK*, and *RPL18A* (IS - specific). The groups ‘Intermediate crucial biomarkers’ contained genes played important roles in H-I disease related biological processes: *CXCL8* (shared), *HBG2*, *GRIN2A*, and *FGFBP1* (PAH - specific), *FAM111B* (HAPC - specific), *C12orf39* and *SLAMF1* (PE - specific). The genes lacking disease-association evidence but with similar characteristics with the above two groups were considered as ‘potential minor-effect biomarkers’: are *SRRM2 - AS1* (shared), *ATP8A1* (PAH - specific), *RXFP1* and *HJURP* (HAPC - specific), *HIST1H1T* (PE - specific). With the development of biological experiments, these intermediate crucial and potential minor-effect biomarkers may be proved to be direct biomarkers in the future. Therefore, these biomarkers may serve as an entry point for subsequent research and are of great significance.

## 1 Introduction

Hypoxic-ischemic (H-I) diseases arise from diverse etiologies and can be categorized into four primary types based on their core pathophysiological characteristics including environment exposure, special physiological stage, and cerebrovascular/cardiovascular conditions. Of which, environment-related disorders, such as high-altitude polycythemia (HAPC), are directly linked to specific environmental factors like hypoxic exposure. Similarly, physiological-stage-related conditions exemplified by eclampsia/preeclampsia (PE) are closely associated with physiological changes during particular life stages, such as pregnancy. Cerebrovascular H-I disorder-including several diseases such as ischemic stroke (IS), hypoxic-ischemic encephalopathy (HIE), and cerebral small vessel disease (CSVD), primarily involve impaired cerebral perfusion or local hypoxia. Cardiovascular H-I disorders, such as pulmonary arterial hypertension (PAH) and myocardial infarction (MI), are characterized by systemic or regional circulatory dysfunction that leads to tissue ischemia and hypoxia. These classifications reflect distinct mechanisms through which hypoxia and ischemia manifest across different organ systems and contexts. All these can lead to severe injuries such as cerebral palsy, brain damage, and even death since it may trigger massive cellular malfunction and cell death (Zhang Z. et al., 2018).

Many researches focused on the common mechanisms of these H-I diseases and found subtle causal relationships among them. Perinatal hypoxia, which was one of the key points of eclampsia, was shown to increase susceptibility to high-altitude polycythemia and attendant pulmonary vascular dysfunction (Julian et al., 2015). Besides, since preeclampsia is a pregnancy-specific disorder resulting in hypertension and multiorgan dysfunction, it was shown to be associated with a 2-fold increased risk in stroke as indicated by a meta-analysis based on 22 studies covering >6.4 million women (Zamudio, 2007). The development of PAH was speculated to be related to polycythemia vera in case reports (Nand and Orfei, 1994; Tachibana et al., 2017). It is possible that the heart and pulmonary vasculature are affected by myeloproliferative process more commonly than is realized. Besides, pulmonary hypertension is a known complication of myeloproliferative neoplasms (MPN) with estimated prevalence as high as 50%. Patients with polycythemia vera (PV) report a wide spectrum of symptoms that significantly overlap with those reported by patients with PAH. Yet, it is not known how PAH affects outcomes and survival in patients with PV(Gazda et al., 2024). Stroke is a major non-cardiac morbidity in patients with pulmonary hypertension as indicated by meta-analysis based on 14 studies including 32,523 participants (Shah et al., 2019). Ischemic stroke is considered as a presenting manifestation of polycythemia vera and validated in a meta-analysis (Burattini et al., 2022).

However, the common mechanisms among these diseases were not clear, especially on H-I level. Previous studies have demonstrated that the phosphatidylinositol-3 kinase (PI3K)/protein kinase B (AKT) signaling pathway, which regulates a wide range of cellular functions, is involved in the resistance response to H-I through the activation of proteins associated with survival and inactivation of apoptosis-associated proteins (Zhang Z. et al., 2018). This indicated that further investigation of biological functions in a systematic way may provide further insights of the potential targets for treating diseases accompanied by H-I.

**FIGURE 1:**
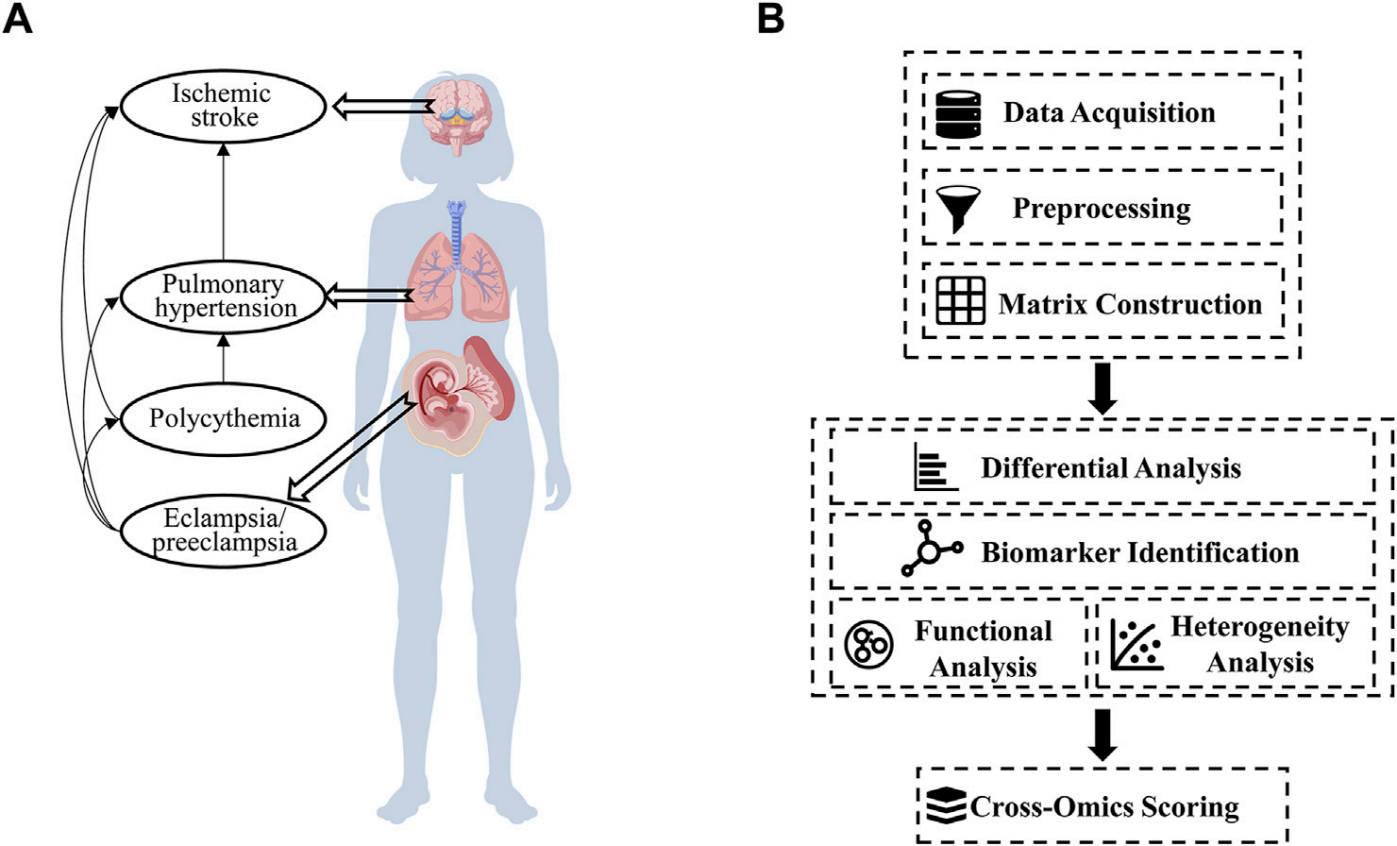
Hypothetical disease-network relationships and the construction of a cross-omics analysis pipeline. **(A)** A proposed network showing the interconnections among ischemic stroke, pulmonary hypertension, polycythemia, and eclampsia/preeclampsia (Created with BioGDP.com) (Jiang et al., 2025). **(B)** The steps of the cross-omics analysis pipeline, including data acquisition, preprocessing, differential analysis, biomarker identification, functional analysis, and cross-omics scoring.

We hypothesize that the observed variations among ischemic-hypoxic diseases may be attributed to differences in vascular perfusion patterns or circulatory mechanisms within the body. To investigate this hypothesis, we selected the four representative diseases mentioned above (PH, HPAC, IS, and PE) (Figure 1A) as model conditions for comparative analysis.

Based on this, we constructed a new H-I disease profile by integrating of multiple transcriptomic datasets. Novel scores were designed for the risk assessment by incorporating intermediate and result data from other omics. This study provides an important theoretical basis for subsequent related research.

## 2 Materials and methods

This work was performed using the following pipeline shown in Figure 1B.

### 2.1 Public H-I datasets

A total of 15 publicly available hypoxia-ischemia (H-I) datasets were retrieved from the Gene Expression Omnibus (GEO) database (Edgar et al., 2002). These datasets include 397 cases, representing samples from four H-I disease groups, and 305 controls, representing samples from the corresponding control groups. The datasets encompass a wide range of experimental data, offering valuable insights into the molecular profiles of H-I diseases. The detailed characteristics of these datasets, including sample features and disease classifications, can be found in Table 1. These datasets provide a comprehensive resource for further investigation into the molecular mechanisms underlying H-I diseases.

**TABLE 1:** Overview of publicly available high-throughput transcriptomic datasets used in this study.

Disease type	GEO accession	Cases	Controls	PMID
Pulmonary Arterial Hypertension (PAH)	GSE254617	96	52	39167456
	GSE168905	12	9	33764154
High-Altitude Erythrocytosis (HAPC)	GSE29977	5	5	\
	GSE145802	70	32	33677043
Ischemic Stroke (IS)	GSE162955	6	6	\
	GSE16561	39	24	20837969; 28446746; 29263821
	GSE202709	12	4	37562178
Eclampsia/Preeclampsia (PE)	GSE30186	6	6	22702245
	GSE10588	17	26	19249095
	GSE24129	8	8	21810232
	GSE25906	23	37	21183218
	GSE43942	5	7	23544093
	GSE4707	10	4	16860862
	GSE44711	8	8	23770704
	GSE75010	80	77	27160201; 28962696; 29187609; 29507646; 30278173; 30312585

### 2.2 Data normalization and statistical analyses

All the datasets were normalized using the following steps.

(1) Feature filter: genes with expression values of zero/missing in more than 75% of the samples were filtered to reduce the impact of low expression levels or extensive missing data.
(2) Missing data imputation: all the left missing values were imputed using zeros.
(3) Min-Max Normalization: each feature was normalized as follows:

$$Normalized\;Value=\frac{Value-\mathrm{Min}}{\mathrm{Max}-\mathrm{Min}}$$

where Min and Max denote the minimum and maximum values of each column, respectively. This normalization method effectively mitigates technical variability between samples, ensuring that the expression data are comparable across different conditions and facilitating accurate downstream analyses.

(4) Disease profile construction: the datasets of a same disease were firstly merged by gene names, row-wise normalization was then performed to standardize the data scale. Different H-I disease datasets (without control) were merged using the same way and batch effect correction was applied using R package sva (Leek et al., 2012) (ComBat function). Outliers with expression values outside the 10th to 90th percentile range were excluded to enhance data quality and improve the accuracy of subsequent analyses.
(5) Differentially expressed gene (DEG) analyses: the expression differences between case and control groups for each gene were analyzed using Wilcoxon Rank-Sum Test. P-values were adjusted using false discovery rate (FDR). Fold change (FC) for each gene was calculated as the ratio of the median and mean expression level between case and control groups. In the analysis process, individual datasets used the median for FC calculation to reduce outlier sensitivity, while integrated datasets employed the mean to improve statistical stability across aggregated samples. All genes were categorized as follows: 1) Upregulated DEGs: genes with FC > 1 and FDR ≤0.05; 2) Downregulated DEGs: genes with FC < 1 and FDR ≤0.05; 3) Non-significant: genes with FC = 1 or FDR >0.05, indicating no significant expression difference between the case and control groups.

### 2.3 Functional analyses

(1) Enrichment analyses: DEGs were subjected to functional enrichment analyses using the clusterProfiler package (Yu et al., 2012) to investigate Gene Ontology (GO) and Kyoto Encyclopedia of Genes and Genomes (KEGG) pathways (Kanehisa and Goto, 2000). A p-value threshold of ≤0.05 was applied to identify significantly enriched GO terms and KEGG pathways, ensuring the statistical significance of the selected functional categories and pathways.
(2) GO term clustering: simplifyGOFromMultipleLists function from the simplifyEnrichment package (Gu and Hübschmann, 2023) was used to find clusters among significantly enriched GO BP terms (p-adj cutoff of 0.05) using org. Hs.eg.db as the annotation database. Clusters with p-adj less than 0.05 were kept.

### 2.4 Cross-omics evaluation risk scores

Six risk scores on different omics levels and one final score were designed to evaluate the roles each gene performed in each disease as follows.

#### 2.4.1 ClinVar score ( $G_{c}$ )

This score is used to assess the potential significance of the association between a gene and a disease, based on the number of variants associated with each gene in the ClinVar database (Landrum et al., 2014). The score is calculated by applying a logarithmic transformation to the variant count (Score) followed by min-max normalization. The formula is as follows:

$$G_{c}=\frac{\log\left(Score+1\right)-\min\left(\log\left(Score+1\right)\right)}{\max\left(\log\left(Score+1\right)\right)-\min\left(\log\left(Score+1\right)\right)}$$

Where Score represents the number of variants in the gene. The logarithmic transformation, $\log\left(Score+1\right)$ , is used to mitigate the impact of extreme values, while min-max normalization ensures that the score falls within the range [0, 1]. Higher scores indicate a greater number of gene variants, suggesting a stronger association with the disease.

#### 2.4.2 pLI score ( $G_{p}$ )

pLI Score ( $G_{p}$ ) (Karczewski et al., 2020) is used to assess the importance of a gene to organismal function, with values ranging from [0, 1]. To ensure compatibility for subsequent multidimensional integration while preserving the characteristics of the original data, a logarithmic transformation combined with a smoothing-based normalization method is applied. The formula is as follows:

$$G_{p}=\frac{\log\left(pLI+\varepsilon \right)-\min\left(\log\left(pLI+\varepsilon \right)\right)}{\max\left(\log\left(pLI+\varepsilon \right)\right)-\min\left(\log\left(pLI+\varepsilon \right)\right)}$$

Where pLI is the raw value, representing the gene’s importance to biological function, and ε is a small constant ( 1e−12 ) added to prevent the calculation of $\log\left(0\right)$ .

#### 2.4.3 Transcriptome score ( $G_{T}$ )

This score is used to assess the importance of a gene at the transcriptome level, calculated as:

$$G_{T}=1-p\_value$$

In the differential expression analysis for each disease, a p_value is calculated for each gene. By transforming the p_value into ${\;G}_{T}$ using the above formula, higher scores indicate greater statistical significance and potential importance of the gene in the disease context. This score provides a quantitative measure for prioritizing genes based on their transcriptomic relevance.

#### 2.4.4 Proteome Score ( $G_{P}$ )

Proteome Score is used to assess the importance of a gene’s corresponding protein in the protein-protein interaction network (PPIN) from STRING (Szklarczyk et al., 2023). The score is based on the degree of each protein in the interaction network, where the degree represents the number of interactions a protein has with other proteins. A higher degree indicates stronger connectivity within the network, suggesting that the protein may play a more crucial role in biological processes. The specific scoring formula is as follows:

$$G_{P}=\frac{\log\left(D_{i}+1\right)-\min\left(\log\left(D_{i}+1\right)\right)}{\max\left(\log\left(D_{i}+1\right)\right)-\min\left(\log\left(D_{i}+1\right)\right)}$$

$D_{i}$ represents the degree of the i -th protein in the network, indicating its interaction count. After applying a logarithmic transformation to the degree values, min-max normalization is used to scale all node degrees to the range [0, 1]. A higher score indicates that the protein has stronger connectivity in the interaction network and may play a key role in processes such as metabolism, biological regulation, or signal transduction.

#### 2.4.5 Metabolome Score ( $G_{M}$ )

Metabolome Score ( $G_{M}$ ) is used to assess the importance of a gene in major metabolic pathways in SMPDB (Frolkis et al., 2010). For each gene in the input file, the associated metabolic pathways are counted, and the most frequent pathway (Pathway) is identified. The score is then assigned based on whether the gene belongs to this Pathway. If the gene is part of the pathway, its score is calculated as the frequency of that Pathway divided by the total number of genes that can successfully match any Pathway. If the gene does not belong to the Pathway, its score is 0. The specific formula is as follows:

$$G_{M}=\frac{n_{dominant}}{N_{total}}$$

Where the most frequent Pathway refers to the pathway that appears most frequently across all genes in the Pathway distribution, and the total number of matching genes refers to the total number of genes that can match any given Pathway. The score naturally falls within the range of [0, 1], with higher scores indicating that the gene may play a key role in the metabolic network, while low scores or a score of 0 suggest a weaker association with major pathways.

#### 2.4.6 Tissue specificity score ( $G_{TS}$ )

We employed the method proposed by Sevahn K. Vorperian et al. for cell lineage tracing of the samples, thereby obtaining the corresponding cell contribution scores (Vorperian et al., 2022). The score TS quantifies the expression specificity of a gene across various tissues by evaluating its overall expression characteristics and distribution in highly specific tissues. The total expression specificity of a gene across all tissues, referred to as total score ( $TS_{total}$ ), is calculated as the sum of its tissue-specific scores ( $TS_{count}$ ):

$$TS_{total}=\sum_{tissue}TS_{score}$$

The number of tissues where the gene exhibits significant specificity ( $TS_{score}>2.5$ ) is represented as number of tissues ( $TS_{count}$ ), calculated as:

$$TS_{count}=\sum_{i=1}^{n}1\left(TS_{score}>2.5\right)$$

To normalize the metrics and ensure consistency across genes, the total score ( $TS_{count}$ ) is scaled to the range [0, 1] using Min-Max Normalization.

Additionally, $TS_{count}$ is normalized by dividing it by a constant factor, $TS_{norm}$ , which represents the total number of tissues in the dataset (32 types).

The combined tissue specificity score ( $G_{TS}$ ) integrates both the normalized total score and the normalized tissue count, capturing the overall expression specificity and the gene’s distribution across highly specific tissues. It is calculated as:

$$G_{TS}=\frac{\frac{TS_{count}}{TS_{norm}}\times \left(TS_{total}-\min\left(TS_{total}\right)\right)}{\max\left(TS_{total}\right)-\min\left(TS_{total}\right)}$$

#### 2.4.7 Final score ( $G_{F}$ )

The integrated final score ( $G_{F}$ ) quantifies the overall importance and multi-omics characteristics of a gene. The weights for each omics layer are determined using principal component analysis (PCA), specifically based on the first principal component (PCA1), which captures the maximum variance in the data. A higher $G_{F}$ indicates a stronger association between the gene and the specific disease, suggesting that the gene may play a more significant role in disease pathology and could serve as a potential biomarker. The weight ( $w_{i}$ ) for each omics layer is calculated as follows:

$$w_{i}=\frac{\left.\left|Loadin\right.g_{i}\right|}{\sum_{j=1}^{n}\left.\left|Loadin\right.g_{j}\right|}$$

In this formula, $\left.\left|Loadin\right.g_{i}\right|$ represents the absolute value of the loading coefficient for the i -th omics layer in PCA1, and $\sum \left.\left|Loadin\right.g_{j}\right|$ is the total sum of the absolute values of all loading coefficients.

The integrated score is then computed as the weighted sum of standardized scores from all omics layers:

$$G_{F}=\sum_{i=1}^{n}w_{i}\times S_{i}$$

$S_{i}$ denotes the standardized score of the i -th omics layer, and $w_{i}$ is the corresponding normalized weight.

#### 2.4.8 Clustering of H-I profile

To explore the relationships between the four diseases based on $G_{F}$ , we performed hierarchical and K-means clustering using the $G_{F}$ matrix (Euclidean distance as the similarity metric and Ward’s method (ward.D2) for linkage). This approach allowed us to identify disease groupings based on shared molecular characteristics, revealing potential similarities and distinctions in their underlying genetic profiles.

#### 2.4.9 Biomarker type classification

All the biomarkers identified were classified into the following 3 types according to the literature search results: 1) direct biomarker: genes played a crucial role in at least one stage of the occurrence, development, and prognosis of a H-I disease; 2) intermediate crucial biomarkers: genes played a key role in a certain crucial biological process related to the occurrence, development, and prognosis of a H-I disease; 3) potential minor - effect biomarkers: genes that currently have no relevant research to confirm their association with the studied disease but possess similar characteristics to the above two types of biomarkers.

## 3 Results

### 3.1 Pan-ischemic hypoxic disease profiles construction

**FIGURE 2:**
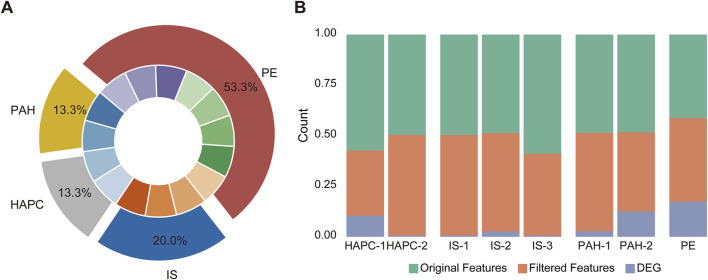
Data distribution and feature processing in the disease datasets. **(A)** The chart illustrates the distribution of diseases across corresponding datasets. The datasets for high-altitude polycythemia (HAPC) are labeled as HAPC-1 (GSE145802) and HAPC-2 (GSE29977), for ischemic stroke (IS) as IS-1 (GSE162955), IS-2 (GSE16561), and IS-3 (GSE202709), for pulmonary arterial hypertension (PAH) as PAH-1 (GSE168905) and PAH-2 (GSE254617), and for eclampsia/preeclampsia (PE) as a combined dataset from GSE75010 along with seven other datasets. **(B)** Feature processing analysis across the datasets. The stacked bar charts depict the proportions of original features, filtered features, and differentially expressed genes (DEGs) for each dataset, including HAPC (HAPC-1 and HAPC-2), IS (IS-1, IS-2, IS-3), PAH (PAH-1 and PAH-2), and PE. These datasets underwent feature filtering and DEG identification, with the processed data shown for each condition.

In this study, we analyzed multi-omics datasets from four H-I diseases. For pulmonary arterial hypertension (PAH), GSE254617 (Hong et al., 2024) and GSE168905 (Li et al., 2021) were included. The raw average number of detectable features was 20,962 ± 3,387, which decreased to 18,723 ± 220 after normalization. 3,544 ± 2,513 DEGs were identified across these datasets. For high-altitude polycythemia (HAPC), GSE29977 and GSE145802 (Tan and Meier-Abt, 2021) were analyzed. A significant difference was observed between these datasets (Figure 2), with the standard deviation of the raw feature counts in GSE145802 being 7,857, which is 2.3 times higher than that of GSE29977. This indicated substantial heterogeneity between datasets. For ischemic stroke (IS), GSE162955, GSE16561 (Barr et al., 2010), and GSE202709 (Kanki et al., 2023) were analyzed. The average number of detectable features before preprocessing was 21,305 ± 4,526, which was reduced to 18,565 ± 1,462 after preprocessing. The average number of DEGs identified across these datasets was 469 ± 384, reflecting a moderate degree of internal variability. For preeclampsia (PE), the data were derived from an integration of datasets from prior studies (Benton et al., 2018). Rigorous preprocessing and analysis of these datasets significantly improved data quality and consistency. Subsequent differential expression analysis of the preeclampsia dataset (comprising 14,651 genomic features) revealed 6,124 statistically significant differentially expressed genes (DEGs). Despite notable differences in the number of detectable features and DEGs across datasets for each disease, the comparison of internal standard deviations provided useful insights into the variability within and among datasets. This variability highlights the inherent heterogeneity and complexity of multi-omics data associated with different H-I diseases.

### 3.2 Shared mechanisms among H-I disease profile

We conducted a comprehensive gene scoring analysis across seven layers for H-I disease profile. This resulted in the construction of the HI-R-DP scoring matrix, consisting of 28 columns, where each column represents scores from a specific layer, and each row corresponds to a gene’s performance across these layers. Subsequently, we calculated the total $G_{F}$ for each disease. Based on the presence of genes in the four diseases, we classified all genes into five groups to explore their commonalities and specificities across multiple diseases.

**FIGURE 3:**
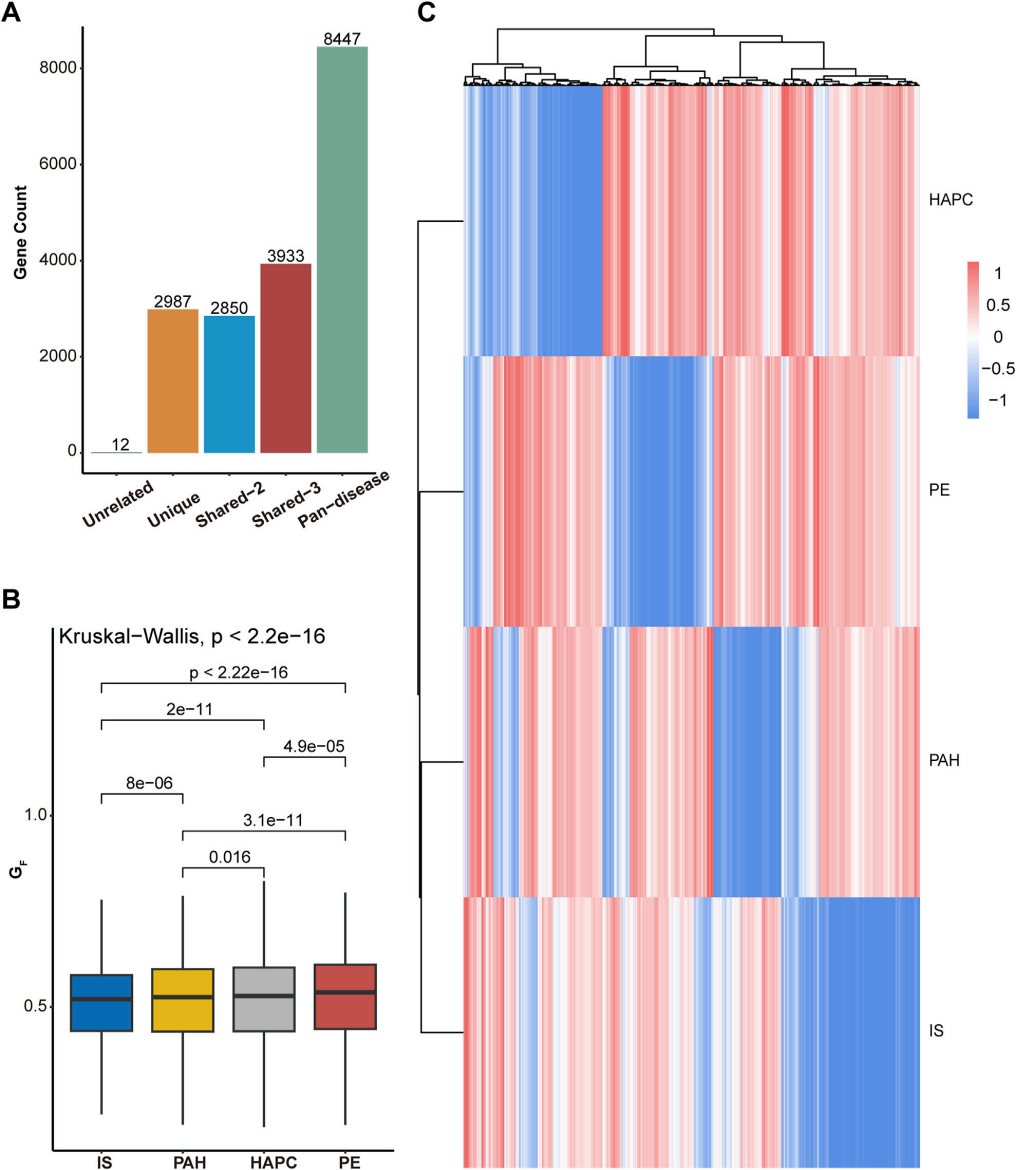
Cross-omics gene scoring and analysis across diseases. **(A)**Distribution of genes into five groups based on cross-omics scoring: “Unrelated” (genes not present in any disease), “Unique” (genes unique to a single disease), “Shared-2” (genes shared between two diseases), “Shared-3” (genes shared between three diseases), and “Pan-disease” (genes present in all diseases). The bar chart shows the number of genes in each group. **(B)** Differences in the total cross-omics scores across the four diseases: ischemic stroke (IS), pulmonary arterial hypertension (PAH), high-altitude polycythemia (HAPC), and eclampsia/preeclampsia (PE). The box plot illustrates the distribution of $G_{F}$ for each disease **(C)** Clustering of total cross-omics $G_{F}$ for each disease. The heatmap shows the clustering of $G_{F}$ , revealing patterns of gene expression across the four diseases.

As shown in Figure 3A, the degree of gene sharing across different diseases varies greatly. The number of pan-disease genes is higher than other types indicating the shared mechanism among H-I diseases. For genes that received scores in all four diseases, the distribution of G_F_ scores was shown in Figure 3B. The Kruskal-Wallis test revealed significant differences in $G_{F}$ across diseases (p < 2.2e-16). Pairwise comparisons showed that the score differences between PAH and HAPC were statistically significant (p = 0.016), suggesting some degree of difference in their gene regulatory patterns, although the difference was smaller compared to other disease pairs. In contrast, PAH exhibited highly significant score differences when compared to PE and IS (p < 1.2e-10), indicating greater molecular regulatory differences among these diseases. Similarly, HAPC also showed significant score differences when compared to PE and IS (p < 1.0e-4), suggesting that key genes in high-altitude polycythemia follow a distinct scoring pattern compared to other diseases. The most pronounced difference was observed between PE and IS (p < 2e-16), implying substantial biological differences in the expression or functional patterns of shared genes between these two diseases. These findings indicate varying degrees of bias in the functional regulation of shared genes across the four diseases, reflecting their unique pathological mechanisms. Although the difference between PAH and HAPC is relatively small, it still suggests potential variations in gene regulation related to vascular adaptation and oxygen supply. In contrast, the strong divergence between PE and IS further supports significant molecular regulatory differences between these conditions.

To further explore the common characteristics of the disease spectrum, we selected genes present in at least three diseases and performed clustering analysis (Figure 3C; Supplementary Figure S1). The analysis revealed that IS and PAH share the most similar gene scoring patterns, suggesting a strong molecular regulatory connection between them. PE displayed a relatively independent gene profile, retaining pregnancy-specific vascular regulation and placental adaptation features, despite some association with IS and PAH. In contrast, HAPC exhibited a gene pattern distinct from the other diseases, likely influenced by mechanisms related to erythropoiesis, oxygen transport, and adaptation to high-altitude hypoxia. This clustering analysis highlights both the similarities and differences between diseases, helping to identify gene modules that may play common roles across multiple diseases. These findings provide new insights for future research on disease mechanisms and potential cross-disease therapeutic strategies.

**FIGURE 4:**
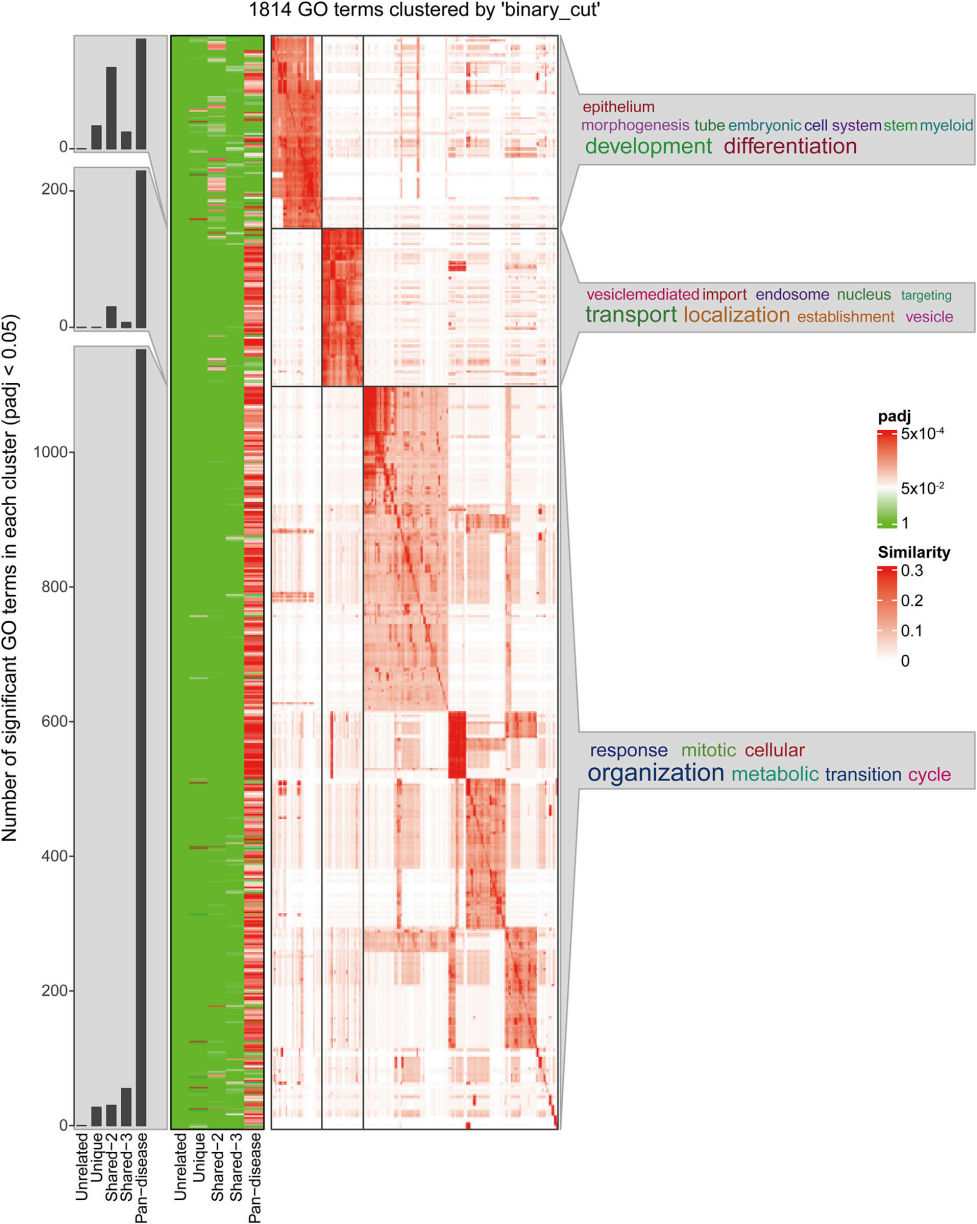
Clustering of GO Biological Process (BP) enrichment results for cross-omics gene groups.

The functional clustering analysis (Figure 4) revealed that genes in the Pan-disease group are widely involved in biological processes such as development and differentiation, substance transport, and cell cycle regulation. This suggests that these genes play crucial roles in maintaining tissue homeostasis, regulating cell proliferation, and adapting to metabolic changes. They may contribute to disease development by modulating vascular development, oxygen transport systems, and cellular adaptation to hypoxic environments. Notably, in ischemia- and hypoxia-related diseases, these genes are likely involved in vascular remodeling, erythropoiesis regulation, and hypoxia-induced signaling pathways, forming a core cross-disease regulatory network.

In contrast, genes in the Unique group are primarily enriched in development- and differentiation-related pathways, indicating their critical roles in tissue-specific angiogenesis, placental function regulation, and local hypoxia adaptation. Many of these genes are involved in stem cell differentiation, embryonic development, and tissue remodeling of specific organs, suggesting their unique functions in pathological conditions such as placental dysfunction, ischemic brain injury, or pulmonary vascular remodeling (see Supplementary Tables S1,S2 for details). The expression patterns of this gene set exhibit stronger tissue specificity across diseases, potentially influencing cellular adaptation and disease progression under local ischemic and hypoxic conditions.

Overall, genes in the Pan-disease group may act as core regulatory factors across multiple diseases, influencing various hypoxia-related pathological processes. In contrast, genes in the Unique group are more associated with disease-specific tissue adaptation mechanisms, particularly in vascular development, erythropoiesis, and local metabolic regulation.

**FIGURE 5:**
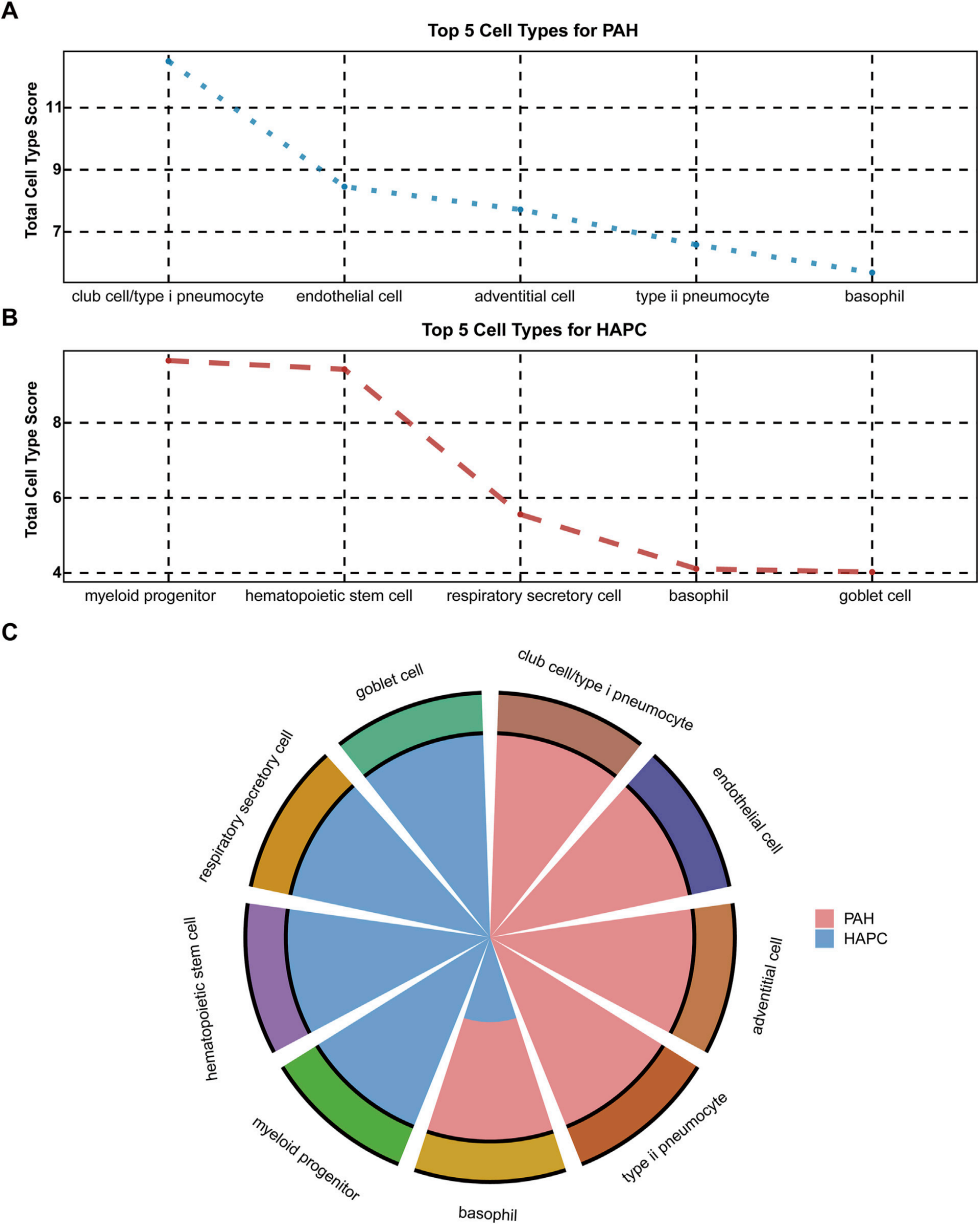
Cell type distribution for PAH and HAPC **(A)** Top 5 cell types for PAH based on total cell type scores **(B)** Top 5 cell types for HAPC based on total cell type scores **(C)** Comparison of cell type distribution for PAH (shown in red) and HAPC (shown in blue).

To conduct an in-depth analysis of the source characteristics of mRNA, this study performed a traceability analysis on the data of HAPC and PAH since the two H-I diseases were shown to be in different clusters (Figure 5C). The top 5 tissues ranked by the cumulative number of all samples of each disease were selected as domain tissue/cell for analysis (results shown in Figures 5A,B). It was found that the mRNA of PAH mainly originated from club cell/type I pneumocyte, endothelial cell, adventitial cell, and type II pneumocyte, which was consistent with clinical knowledge. For HAPC samples, the cells were mostly derived from myeloid progenitor, hematopoietic stem cell, etc., also in line with clinical understanding. Among them, the common cell was basophil, and its correlation with the two diseases had been confirmed by previous studies (Ni et al., 2022; Yuen et al., 2025). Besides, the immune response following hypoxia - ischemia events in various diseases indicated its downstream events in H-I diseases (Eltzschig and Eckle, 2011; Albertsson et al., 2014).

**FIGURE 6:**
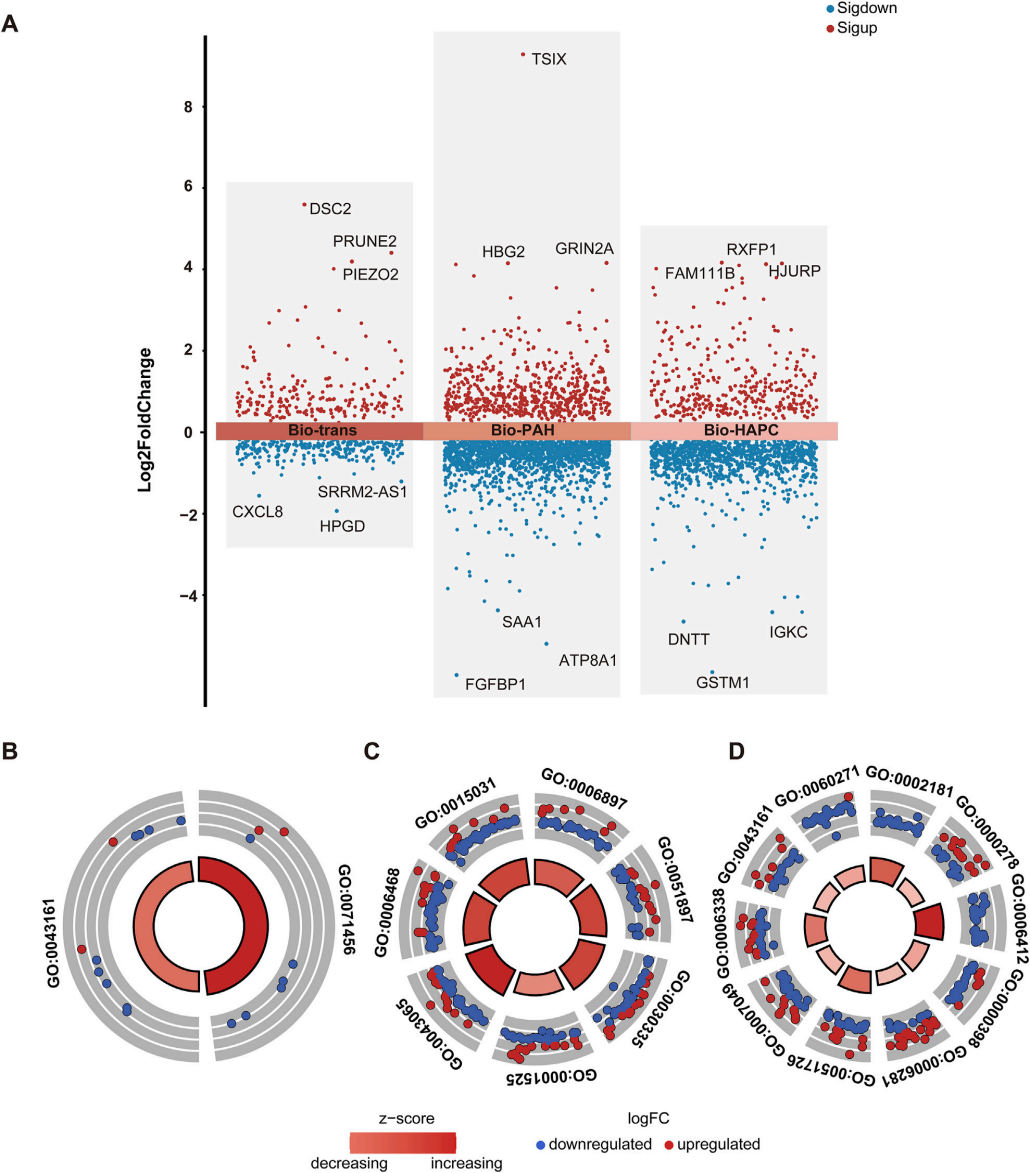
Differential gene distribution and enrichment analysis of Bio-trans, Bio-PAH, and Bio-HAPC gene sets **(A)** Volcano plot of differential gene expression for Bio-trans, Bio-PAH, and Bio-HAPC, with significant genes (p < 0.05) highlighted in red (upregulated) and blue (downregulated) **(B)** GO BP enrichment analysis for the Bio-trans gene set, shown in a circular plot **(C)** GO BP enrichment analysis for the Bio-PAH gene set **(D)** GO BP enrichment analysis for the Bio- HAPC gene set.

923 common genes of HAPC and PAH were identified as raw trans-biomarkers. After removing genes overlapping with those specific to IS, PE, and unique disease-specific genes, a final set of 495 trans-biomarkers (herein denoted as Bio-trans) was obtained. These genes showed differential expression in both HAPC and PAH but not in other diseases, suggesting their involvement in shared hypoxia-related molecular mechanisms. The top genes obtained were listed in Table 2 (Figure 6A). *DSC2* was proven to be directly associated with myocardial function under the regulation of Cycloastragenol (Ren et al., 2020). Hypoxia - ischemia was considered as secondary injury in spinal cord injury (SCI) (Tator and Fehlings, 1991). The increased expression of *Piezo2* was associated with poorer urodynamic parameters in SCI mice (Gotoh et al., 2022). *Piezo2* played a key role in PAH, and its deficiency was shown to be associated with PAH by impairing NO synthesis and inducing EndMT (Tian et al., 2022; Wei et al., 2025). There were no relevant literature on polycythemia, so the results of this study suggested a possible relationship. The results of our study also showed upregulation, which was consistent. Downregulation of *HPGD* could improve the proliferation activity, reduce apoptosis, and enhance adhesion and angiogenesis in endothelial cells (ECs), thus promoting the occurrence and development of hypoxic pulmonary hypertension (He et al., 2023). The results of our study also showed downregulation, which was consistent. There was no previous research on its relationship with polycythemia, so our results suggested a possible relationship. *CXCL8* was a promising biomarker of inflammation - sensitized hypoxia, as validated in an inflammation - sensitized hypoxia - ischemia model (Lingam et al., 2021). Currently, there were no studies on *PRUNE2* and *SRRM2-AS1* in the above - mentioned diseases, so these two genes could be potential new markers. The key themes across these genes highlight their roles in vascular remodeling, inflammation, and metabolic dysregulation. Of which, *HPGD* and *PIEZO2* are central to vascular dysfunction, with *HPGD* downregulation driving angiogenesis and pulmonary hypertension via enhanced endothelial proliferation and reduced apoptosis, while *PIEZO2* deficiency impairs NO synthesis and promotes EndMT, exacerbating PAH and SCI-related complications. *CXCL8* emerges as a critical biomarker linking inflammation-sensitized hypoxia to tissue injury, as validated in ischemic models. Meanwhile, *SRRM2-AS1* and PRUNE2 represent understudied candidates with potential relevance to vascular or metabolic disorders, warranting further investigation. *HPGD’s* angiogenic effects and *PIEZO2*’s disruption of NO signaling indirectly implicate these genes in redox imbalance. Collectively, these genes collectively underscore the interplay between vascular integrity, inflammation, and metabolic adaptation in diseases like PAH, SCI, and ischemia, with emerging roles for non-coding RNAs and novel targets in therapeutic strategies.

**TABLE 2:** Top 3 upregulated and downregulated differentially expressed genes in Bio-trans, Bio-HAPC, and Bio-PAH gene sets.

geneID	log2FC	p_value	p_val_adj	cluster	label	biomarker type
*DSC2*	5.025496	1.483261e-03	2.302198e-02	Bio-trans	Sigup	Intermediate crucial
*PRUNE2*	3.954060	1.361934e-03	2.204804e-02	Bio-trans	Sigup	Potential minor-effect
*PIEZO2*	3.762164	6.547431e-06	1.232190e-03	Bio-trans	Sigup	Direct
*TSIX*	8.353926	2.502102e-04	5.617039e-03	Bio-PAH	Sigup	Direct
*HBG2*	3.730207	3.507502e-05	1.556768e-03	Bio-PAH	Sigup	Intermediate crucial
*GRIN2A*	3.723723	2.738851e-05	1.309408e-03	Bio-PAH	Sigup	Intermediate crucial
*RXFP1*	3.736920	2.739276e-12	3.711719e-08	Bio-HAPC	Sigup	Potential minor-effect
*FAM111B*	3.719850	1.411383e-03	2.249318e-02	Bio-HAPC	Sigup	Intermediate crucial
*HJURP*	3.701026	2.676796e-02	1.344701e-01	Bio-HAPC	Sigup	Potential minor-effect
*HPGD*	−1.767203	1.583123e-06	6.919780e-04	Bio-trans	Sigdown	Direct-PAH
*CXCL8*	−1.429028	6.737854e-03	5.749240e-02	Bio-trans	Sigdown	Intermediate crucial
*SRRM2-AS1*	−1.112642	1.603463e-03	1.878793e-02	Bio-trans	Sigdown	Potential minor-effect
*FGFBP1*	−5.406030	4.154511e-08	1.369221e-05	Bio-PAH	Sigdown	Intermediate crucial
*ATP8A1*	−4.712998	4.167374e-02	1.481924e-01	Bio-PAH	Sigdown	Potential minor-effect
*SAA1*	−3.971495	1.168539e-12	4.525168e-09	Bio-PAH	Sigdown	Direct
*GSTM1*	−5.339262	1.234758e-06	6.141446e-04	Bio-HAPC	Sigdown	Direct
*DNTT*	−4.219855	7.413852e-04	1.538403e-02	Bio-HAPC	Sigdown	Direct
*IGKC*	−4.012964	6.380791e-06	1.232190e-03	Bio-HAPC	Sigdown	Direct
*LEP*	0.7903525	2.316661e-31	3.394141e-27	Bio-PE	Sigup	Direct
*SERPINA3*	0.5813629	6.368638e-17	1.003300e-14	Bio-PE	Sigup	Direct
*ARHGEF4*	0.4219916	5.946371e-17	9.469596e-15	Bio-PE	Sigup	Direct
*HIST1H1T*	−0.4715339	3.492409e-09	9.493003e-08	Bio-PE	Sigdown	Potential minor-effect
*C12orf39*	−0.4697635	2.773734e-15	2.745810e-13	Bio-PE	Sigdown	Intermediate crucial biomarkers
*SLAMF1*	−0.3846013	1.850801e-18	4.108497e-16	Bio-PE	Sigdown	Intermediate crucial biomarkers
*CD3D*	−0.7202023	1.834311e-02	3.822185e-01	Bio-IS	Sigdown	Direct
*ITK*	−0.6823456	2.939615e-02	3.822185e-01	Bio-IS	Sigdown	Direct
*RPL18A*	−0.6412929	2.381060e-02	3.822185e-01	Bio-IS	Sigdown	Direct

The expression profiles of Bio-trans genes were analyzed using the GTEx (Genotype-Tissue Expression) (GTEx Consortium, 2013) data to determine their cellular and tissue origins Analysis revealed distinct expression patterns across multiple genes. Of which, the *HPGD* gene was predominantly expressed in vascular endothelial cells of the lung (detected in cells: 9.20%) and alveolar macrophages (detected in cells: 17.95%). Similarly, *CXCL8* exhibited primary expression in club epithelial cells of the lung (detected in cells: 3.18%) and alveolar macrophages (detected in cells: 2.05%). *SRRM2-AS1* demonstrated tissue-specific expression in lymphatic endothelial cells of mammary tissue (detected in cells: 3.36%) and basal epithelial cells of the lung (detected in cells: 3.52%). Notably, *DSC2* showed broad tissue distribution, with high expression in suprabasal epithelial cells of the esophageal mucosa (detected in cells: 40.52%), alveolar type I epithelial cells of the lung (detected in cells: 1.44%), and both cytoplasmic (detected in cells: 8.86%) and non-cytoplasmic (detected in cells: 7.98%) cardiac myocytes in the left ventricle. *PRUNE2* was enriched in ciliated epithelial cells of the lung (detected in cells: 20.31%) and adipocytes of the left ventricular heart tissue (detected in cells: 23.32%). Strikingly, *PIEZO2* displayed dominant expression in lung fibroblasts (detected in cells: 47.56%), with additional activity in lung lymphatic (detected in cells: 39.82%) and vascular endothelial cells (detected in cells: 7.13%).

GO biological process (BP) enrichment analysis of these trans - biomarkers identified two significantly enriched pathways: cellular response to hypoxia (GO:0071456) and proteasome-mediated ubiquitin-dependent protein catabolic process (GO:0043161) (Figure 6B). Hypoxia is a well - known trigger for PAH, with mitochondrial dysfunction and oxidative stress playing critical roles in its pathogenesis (Ahmed et al., 2024). Similarly, in polycythemia, hypoxia resulting from pulmonary vascular abnormalities may lead to disease progression (Lertzman et al., 1964). Alterations in the ubiquitin-proteasome system (UPS) due to hypoxia have been implicated in PAH (Wade et al., 2018), and dysfunction of the UPS has also been linked to abnormal red blood cell production in polycythemia (Meyer et al., 2007). Collectively, these findings suggest that hypoxia - related molecular mechanisms underlie both PAH and polycythemia.

### 3.3 Disease-specific biomarkers and function

#### 3.3.1 PAH - Specific biomarkers

For PAH-specific biomarkers (Bio-PAH), 2,242 genes were obtained after excluding genes overlapping with those of HAPC, IS, and PE. The top genes obtained were listed in Table 2 (Figure 6A). These genes were divided into the following types Knockdown of *TSIX* could improve functional recovery and attenuate the inflammation response and cell apoptosis via the *miR - 30a/SOCS3* axis (Pan et al., 2024). It could also aggravate spinal cord injury (SCI) by regulating the *PI3K/AKT* pathway via the *miR-532-3p/DDOST* axis (Dong et al., 2023). Besides, upregulation of *TSIX* could partially explain the sexual dimorphism of female pulmonary artery endothelial cells (ECs). This is consistent with our results, as this gene was also upregulated in our study (Carman et al., 2024). Regarding *HBG2*, although it is related to hypoxia, no direct research on its association with PAH has been found. Hypoxia can induce the transcription of γ - globin genes, including *HBG2*, by stabilizing HIF1α, enabling the HIF1α - HIF1β heterodimers to bind to the DNA elements of the *BGLT3* gene downstream of *HBG2* (Feng R. et al., 2022). *GRIN2A* is related to myocardial infarction, yet no direct research on its connection with PAH has been reported. *GRIN2A* was considered a candidate biomarker of acute myocardial infarction (Wu et al., 2022), which is closely related to PAH (Møller et al., 2005). Pathway analysis shows an association between *FGFBP1* and hypertension (Tomaszewski et al., 2011). However, in this previous study, *FGFBP1* was upregulated, while in our study, it was downregulated. This difference might be attributed to the distinction between hypertension and pulmonary hypertension. *SAA1* was shown to be related to the pathogenesis of idiopathic pulmonary arterial hypertension, regardless of sex differences (Xu et al., 2021). Currently, no research on the relationship between *ATP8A1* and pulmonary hypertension has been found. Given its minor effect, we speculate that this gene may function jointly with other genes.

GO BP enrichment analysis identified seven significantly enriched pathways (Figure 6C). These pathways include endocytosis (GO:0006897), positive regulation of the *PI3K/Akt* signaling pathway (GO:0051897), angiogenesis (GO:0001525), positive regulation of cell migration (GO:0030335), positive regulation of the apoptotic process (GO:0043065), protein transport (GO:0015031), and protein phosphorylation (GO:0006468). Disrupted endocytosis contributes to PAH progression by affecting surface protein internalization and trafficking (Chichger et al., 2019). The *PI3K/Akt* signaling pathway has been implicated in hypoxic PAH, with studies showing that improvements in this pathway can mitigate endothelial and mitochondrial dysfunction (Shi et al., 2023). Vascular remodeling, driven by angiogenesis, cell migration, and apoptosis, is a hallmark of PAH and involves processes such as cell hypertrophy, proliferation, and migration (Tajsic and Morrell, 2011). Additionally, protein phosphorylation plays an essential role in cellular signaling, with alterations in phosphorylation pathways contributing to PAH pathogenesis (Zhang J. et al., 2018; Li et al., 2024).

#### 3.3.2 HAPC-specific biomarkers

For HAPC-specific biomarkers (Bio-HAPC), 1,425 genes were obtained after excluding genes overlapping with those of PAH, IS, and PE. The top genes obtained were listed in Table 2. A significant reduction in *RXFP1* expression was observed in the ischemic myocardium (Gao et al., 2019). Mutations in the *FAM111B* gene may predict the severity of pulmonary fibrosis and a poor prognosis (Arowolo et al., 2022). Pulmonary fibrosis may be one of the causes of polycythemia (Ghosh et al., 2021). In the Jordanian population, the *GSTM1* null genotype alone and in combination with the *CYP1A1* m1 genotype may be predisposing risk factors for polycythemia vera (Naffa et al., 2012). *DNTT* was found to be downregulated in polycythemia vera and considered as a diagnostic marker (Baumeister et al., 2021). *IGKC* was downregulated in polycythemia vera (Gangaraju et al., 2020). Currently, there is no research on the relationship between *HJURP* and polycythemia.

GO BP enrichment analysis revealed 10 significantly enriched pathways, all related to the cell cycle (Figure 6D). These findings reflect the hyperproliferative characteristics of polycythemia, highlighting the role of cell cycle regulation in the development and progression of the disease (Lertzman et al., 1964). Dysregulation of the cell cycle may drive excessive red blood cell production, contributing to the pathological mechanisms underlying HAPC.

#### 3.3.3 PE-specific biomarkers

For PE-specific biomarkers (Bio-PE), 3496 genes were obtained after excluding genes overlapping with those of PAH, HAPC, and IS. The top genes obtained were listed in Table 2 (Figure 6A). *LEP* and *ARHGEF4*, involved in metabolic and hypoxia/angiogenesis pathways, are shown to be upregulated (in accordance with our result) in placentas from severe preeclampsia (sPE) patients across ancestries, suggesting its contribution to the pathophysiology of preeclampsia (Aisagbonhi et al., 2023). The *SERPINA3* gene is identified as a key diagnostic biomarker for preeclampsia, showing differential expression in affected placentas and associations with immune cell infiltration (Yang et al., 2022).

The *HIST1H1T* gene plays a critical role in sperm maturation by facilitating histone-to-protamine replacement in spermatocytes, and its disruption in double-knockout mouse models synergizes with other genes (e.g., Mcsp) to impair sperm function (e.g., morphology, motility, fertilization) and severely compromise fertility, indirectly impacting embryonic development by disrupting successful fertilization (Nayernia et al., 2003). The *C12orf39* gene exhibits differential expression in placental tissue from mothers with antenatal depression and those using antidepressants during pregnancy, suggesting its potential role in placental dysfunction associated with maternal mental health or pharmacological exposure (Olivier et al., 2014). The *SLAMF1* gene is downregulated in placental microvascular endothelial cells from severe intrauterine growth restriction (IUGR) cases compared to controls, and its expression differences are confirmed in placental tissue microarray analyses, suggesting its role in vascular dysfunction in pregnancy complications like PE (Dunk et al., 2012).

#### 3.3.4 IS-specific biomarkers

For IS-specific biomarkers (Bio-IS), 405 genes were obtained after excluding genes overlapping with those of PAH, HAPC, and PE. The top genes obtained were listed in Table 2. *CD3D*, *ITK* were considered to be IS biomarkers by former researches (Feng S. et al., 2022; Wei et al., 2023; Wang et al., 2024). The *RPL18* gene is a key mediator in stroke pathophysiology, as its dysregulation is linked to cerebral ischemia, and its restoration via traditional medicines (e.g., BYHW, NXT, YYTN) improves outcomes by modulating the gut microbiota-brain axis and suppressing neuroinflammation (microglia/astrocyte hyperactivation) (Yin et al., 2022).

## 4 Discussion

In this study, we devised a novel risk assessment method to infer the shared features among four H-I diseases in the presence of heterogenous omics data. Traditional bioinformatics analyses were performed to construct a H-I disease profile based on transcriptomic data since mRNAs responded promptly to abnormal physiological states in the human body and were easy to detect. Considering the lack of large scale high-quality multi - omics data that specifically target the same disease, population, and tissues, we exploited several public databases/datasets to design various risk assessment scores. A final new evaluation score was designed to integrate evaluation information from genomics, proteomics, and metabolomics in previous researches. The similarity and differences among these H-I diseases were then analyzed on both feature molecules and functional levels.

We divided all the genes in pan disease profile into groups. The ‘pan-disease genes’ were shown to play a central role in the occurrence and development of multiple diseases. Specifically, they may influence the pathological processes of these diseases by regulating some common biological processes, such as angiogenesis, hypoxic response, and cell proliferation. Further analysis may reveal the common regulatory roles of these genes in different diseases and provide clues for cross-disease biomarkers. Compared with this, unique group genes are crucial for disease - specific tissue adaptation, especially in vascular and metabolic aspects, which advances our understanding of disease genetic mechanisms.

The four diseases investigated in this study present distinct clinical manifestations. However, at the molecular level, all of them are associated with ischemia or hypoxia. Besides, these features may also be explained from the body’s circulatory system. IS is a typical systemic circulation disease. It occurs when there is an obstruction in the blood vessels of the brain, which are part of the systemic circulation network. The lack of blood supply to the brain tissue due to blockages in arteries like the carotid artery or its branches leads to ischemic injury. The pathophysiological mechanisms involve factors such as thrombosis formation, embolism, and atherosclerotic plaque rupture within the systemic arterial system, with little direct connection to the pulmonary circulation in its primary etiology. PE is a condition unique to pregnancy, mainly affects the systemic circulation. It is characterized by systemic small - vessel vasospasm, endothelial cell injury, and subsequent organ dysfunction. The placenta, which is part of the maternal - fetal circulatory system (a specialized part of the systemic circulation during pregnancy), plays a crucial role. The abnormal placentation and reduced placental perfusion can trigger a cascade of systemic responses, leading to hypertension, proteinuria, and potential involvement of multiple organs such as the kidneys, liver, and heart. In severe cases, PE can also impact the pulmonary circulation, causing pulmonary edema, indicating its complex relationship with both circulatory systems. HAPC is closely related to the pulmonary circulation. In individuals with HAPC, the body’s adaptation to the hypoxic environment at high altitudes leads to an increase in red blood cell production. This process is mainly regulated within the context of the pulmonary circulation as the lungs are the primary organs sensing the low - oxygen condition. The subsequent elevation in hematocrit aims to enhance oxygen - carrying capacity, but it also brings about changes in the pulmonary vascular bed, such as increased blood viscosity, which may affect pulmonary hemodynamics. PAH has a complex relationship with both the systemic and pulmonary circulations. Initially, it is considered a pulmonary circulation disorder, where abnormal remodeling of the pulmonary arteries occurs, leading to increased pulmonary vascular resistance. This results in elevated pulmonary arterial pressure and impaired right - heart function. However, systemic factors cannot be ignored, which is similar to that of IS. For example, systemic inflammatory cytokines can be released into the bloodstream and reach the pulmonary vasculature, promoting endothelial dysfunction and smooth muscle cell proliferation in the pulmonary arteries. This was also validated in this study (See Figure 3C for details).

In our results, we found PAH and HAPC were more similar to the other two diseases which may be cause partly due to their involvement in pulmonary circulation especially in hypoxic response and vascular regulation. The significant differences between PAH and PE, IS validated that the differences of these diseases may be caused by the interactions of human body systems based on circulation systems. As a pregnancy-related disease, PE may be influenced by unique vascular regulatory mechanisms during pregnancy. IS, on the other hand, involves ischemic injury to the nervous system, fundamentally different from the molecular mechanisms of PAH and HAPC. Therefore, further investigation of the differences in gene regulation among these three diseases will contribute to uncovering their underlying biological disparities. To illustrate the molecular features in details, we traced the mRNA sources in HAPC and PAH and basophil was found to be the common cell type among the top - 5 - ranked cells, which reflected the downstream role of immune response in H - I diseases.

Of the 18 top biomarkers in each group, the number of direct, intermediate crucial, and potential minor - effect biomarkers were 7, 6, and 5, respectively. These indicated our novel method can effectively identify genes related to H - I diseases that have been confirmed in previous experiments, thus validating the effectiveness of our risk assessment score. In summary, the “Bio - HAPC” category has the largest number of direct biomarkers, the “Bio - PAH” category has the largest number of intermediate crucial biomarkers, and the “Bio - trans” category has the largest number of potential minor - effect biomarkers. This indicates that the current types of omics data (in this study, GWAS results related to HAPC could not be obtained from public databases) can basically meet the research needs for HAPC. Regarding PAH, perhaps due to the complexity of its pathogenesis, most of the top - ranked genes are involved in biological processes closely related to the occurrence of PAH, which also validates the effectiveness of this method to some extent. Interestingly, there are currently no other systematic evaluation studies on H - I diseases. Therefore, the potential minor - effect biomarkers identified in this study can serve as an entry point for subsequent research and are of great significance. However, with the development of biological experiments, these intermediate crucial and potential minor-effect biomarkers may be proved to be direct biomarkers in the future.

The interplay among the four H-I diseases underscores a complex network of shared mechanisms and bidirectional risks. HAPC, characterized by hypoxia-induced erythrocytosis, substantially increases blood viscosity and causes vascular endothelial dysfunction, thereby elevating the risk of developing PAH. Moreover, HAPC contributes to cerebrovascular events through thrombosis and microvascular ischemia. PAH, in turn, exacerbates cerebral hypoperfusion, creating an indirect link to IS via shared hypoxic and pro-thrombotic pathways. PE induces systemic endothelial dysfunction. This not only predisposes patients to cerebrovascular complications but also heightens the risk of cardiovascular events. Significantly, PAH is a well - recognized complication of severe PE, highlighting a direct pathophysiological overlap between the two conditions. Conversely, chronic hypoxic states, such as those associated with HAPC, may exacerbate PE - like symptoms in pregnant individuals at high altitudes due to shared mechanisms of placental ischemia and systemic inflammation. These cross - disease relationships, where one condition can both contribute to and be a consequence of others, underscore the necessity for integrated research frameworks and holistic management strategies. Such approaches are essential for effectively addressing the overlapping pathophysiological cascades in H-I disorders.

The bidirectional risks between H-I diseases necessitate proactive clinical strategies. When a patient presents with an H-I condition such as PE/eclampsia, clinicians should initiate aggressive acute - phase management. This includes strict blood pressure control, administration of anticonvulsants (e.g., magnesium sulfate), and timely delivery. Concurrently, comprehensive diagnostic evaluations should be performed. Brain imaging (MRI/CT) is crucial for detecting silent cerebral ischemia or hemorrhage. Echocardiography and right heart catheterization are essential for screening PAH, while blood tests (e.g., D - dimer, blood viscosity) help assess the risk of thrombosis and HAPC. Long - term management requires lifelong cardiac follow - up for PE survivors. Serial echocardiograms can facilitate early detection of PAH. For PE/HAPC patients, stroke risk evaluations using carotid ultrasound and cognitive screening are essential. In individuals with chronic hypoxia or PAH, vigilance against polycythemia - driven thromboembolism is necessary. Multidisciplinary, integrated care is crucial to mitigate comorbidities and improve patient outcomes.

The primary limitations of this study stem from the restricted scope of data sources and sample diversity. First, the datasets relied upon in this analysis were predominantly based on transcriptomic profiles, while proteomic, metabolomic, or epigenetic data were either derived from intermediate analytical outputs or secondary data from public repositories. This limited integration of multi-omics layers constrained our ability to systematically dissect multiscale molecular mechanisms underlying hypoxia-ischemia (H-I) disorders. For instance, the lack of direct experimental data linking gene expression changes to protein post-translational modifications or metabolic pathway perturbations hindered a comprehensive understanding of disease-driven molecular networks. Second, the sample size and ethnic representativeness were insufficient to ensure generalizability. Most data originated from single-center studies or populations of specific ethnic backgrounds (e.g., European ancestry), lacking coverage of diverse geographic, genetic, and environmental exposure groups. This limitation may have led to the omission of critical gene-phenotype associations (e.g., functional racial differences in genes like *HIST1H1T*, *RPL18A*, or *PIEZO2*) and limited our capacity to explore race-specific risk profiles or therapeutic response heterogeneity. Additionally, the absence of longitudinal dynamic data restricted insights into temporal disease progression. H-I pathologies involve dynamic transitions from acute ischemic insults to chronic vascular remodeling, but static datasets cannot capture the temporal evolution of molecular markers (e.g., the timing of hemodynamic changes relative to gene expression shifts).

Despite these constraints, the methodological framework developed in this study is inherently scalable. Future research leveraging large-scale, integrated multi-omics data and ethnically diverse, prospective cohorts could significantly expand our findings by: (1) integrating transcriptomic, proteomic, and epigenetic data to unravel hierarchical regulatory mechanisms; (2) comparing cross-ethnic datasets to identify universal disease-driving modules and race-specific modifiers; and (3) analyzing time-series data to map the spatiotemporal dynamics of key pathways (e.g., endothelial dysfunction-inflammation-vascular remodeling axes).

## Data Availability

Publicly available datasets were analyzed in this study. This data can be found here: The datasets analyzed in this study are publicly available in the Gene Expression Omnibus (GEO) repository. The corresponding accession numbers and direct links are: PAH: GSE254617 (https://www.ncbi.nlm.nih.gov/geo/query/acc.cgi?acc=GSE254617), GSE168905 (https://www.ncbi.nlm.nih.gov/geo/query/acc.cgi?acc=GSE168905) HAPC: GSE29977 (https://www.ncbi.nlm.nih.gov/geo/query/acc.cgi?acc=GSE29977), GSE145802 (https://www.ncbi.nlm.nih.gov/geo/query/acc.cgi?acc=GSE145802) IS: GSE162955 (https://www.ncbi.nlm.nih.gov/geo/query/acc.cgi?acc=GSE162955), GSE16561 (https://www.ncbi.nlm.nih.gov/geo/query/acc.cgi?acc=GSE16561), GSE202709 (https://www.ncbi.nlm.nih.gov/geo/query/acc.cgi?acc=GSE202709) PE: GSE75010 (https://www.ncbi.nlm.nih.gov/geo/query/acc.cgi?acc=GSE75010).
