# Supplementary material for: Shared genetic features inference among hypoxia-ischemia diseases in the presence of heterogenous omics data based on a novel risk assessment method
Source: Front Genet. 2025 Apr 28;16:1587854. doi: 10.3389/fgene.2025.1587854 (PMC12066567; doi:10.3389/fgene.2025.1587854)
Supplement: Supplementary file 1 [file Table2.docx]

**
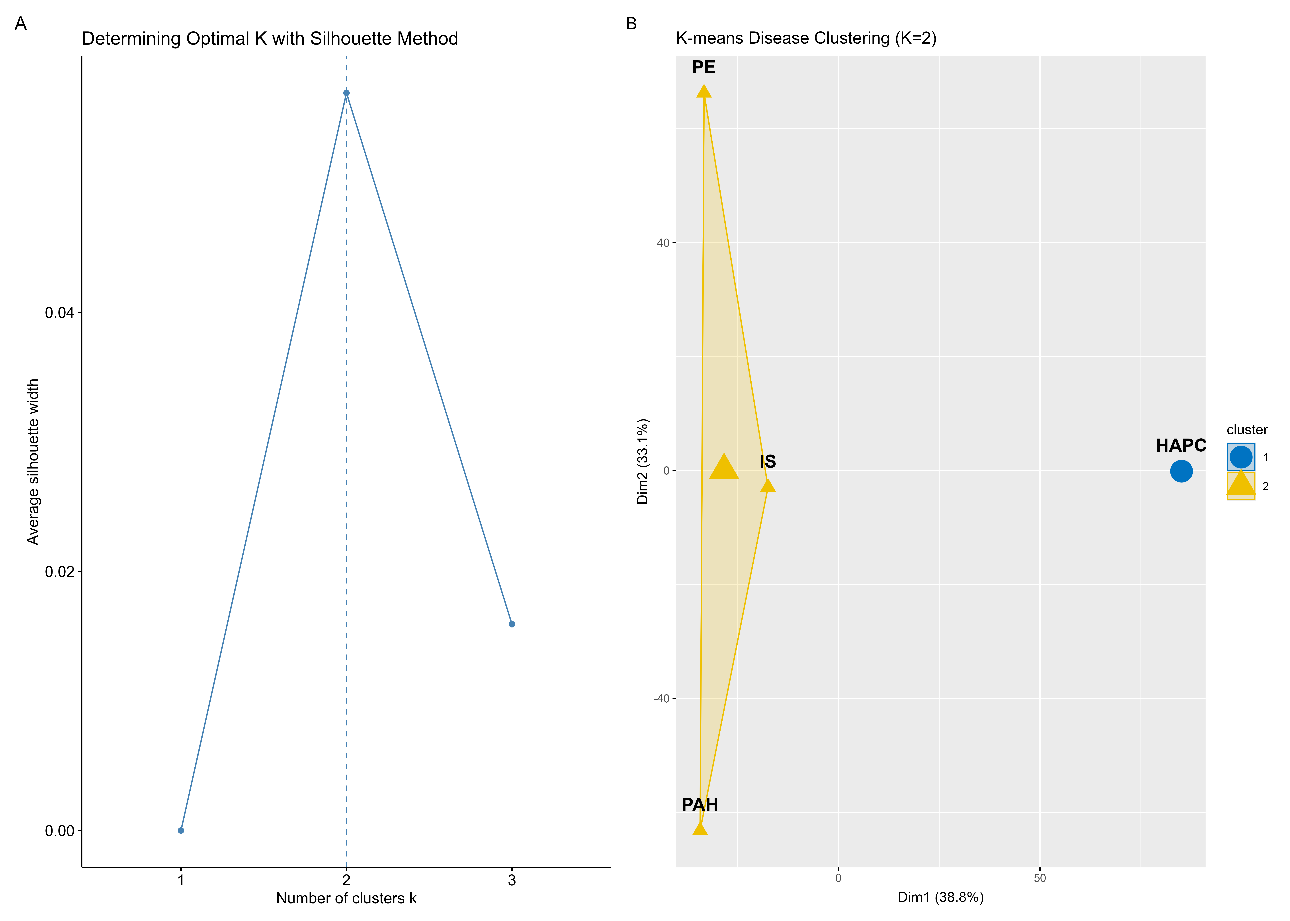
**

**FIGURE S1:** **K-means Clustering Analysis of Disease Relationships**

(A) Determination of optimal cluster number using silhouette method.

(B) K-means clustering of diseases based on $G_{F}$. The diseases are clustered into two distinct groups: HAPC forms a separate cluster (Cluster 1), while PAH, PE, and IS group together (Cluster 2).
